# Advances in Microalgae-Derived Phytosterols for Functional Food and Pharmaceutical Applications

**DOI:** 10.3390/md13074231

**Published:** 2015-07-09

**Authors:** Xuan Luo, Peng Su, Wei Zhang

**Affiliations:** 1 Flinders Centre for Marine Bioproducts Development, Flinders University, Adelaide, SA 5042, Australia; E-Mails: luo0033@flinders.edu.au (X.L.); peng.su@flinders.edu.au (P.S.); 2 Department of Medical Biotechnology, School of Medicine, Flinders University, Adelaide, SA 5042, Australia

**Keywords:** microalgae, lipids, phytosterols, functional food, pharmaceuticals

## Abstract

Microalgae contain a variety of bioactive lipids with potential applications in aquaculture feed, biofuel, food and pharmaceutical industries. While microalgae-derived polyunsaturated fatty acid (PUFA) and their roles in promoting human health have been extensively studied, other lipid types from this resource, such as phytosterols, have been poorly explored. Phytosterols have been used as additives in many food products such as spread, dairy products and salad dressing. This review focuses on the recent advances in microalgae-derived phytosterols with functional bioactivities and their potential applications in functional food and pharmaceutical industries. It highlights the importance of microalgae-derived lipids other than PUFA for the development of an advanced microalgae industry.

## 1. Phytosterols: Chemistry, Origin and Applications

### 1.1. Chemistry of Phytosterols 

**Figure 1:**
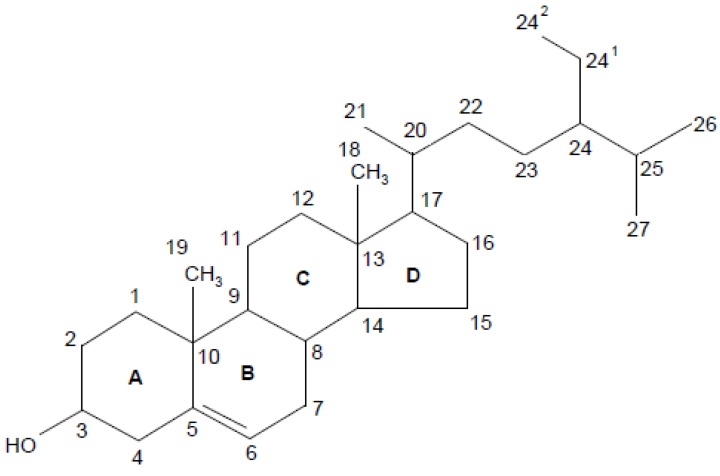
Nomenclature of phytosterols. Numbering follows the International Union of Pure and Applied Chemistry and International Union of Biochemistry 1989 recommendations with modifications [1].

There has been no standardisation of phytosterol nomenclature. The most commonly adopted phytosterol nomenclature is in the International Union of Pure and Applied Chemistry and International Union of Biochemistry recommendations 1989 [1] (Figure 1). As shown, phytosterols are characterised by a tetracyclic cyclopenta (α) phenanthrene structure (ring A, B, C and D) and an aliphatic side chain (R) at C_17_ of ring D. They are amphiphilic due to the polar hydroxyl group (OH) at C_3_ of ring A and have a non-polar structure for the rest. In most cases, phytosterols have a double bond between C_5_ and C_6_ and methyl groups at C_10_ and C_13_. From domain D, the length, position of double bond, absence or presence of a methyl or ethyl group, saturation and stereochemistry of the C_24_ alkyl side chain are critical to intermolecular contacts and function of phytosterols [2]. Phytosterols may also be represented using C*_x_*Δ*_y_* where *x* indicates the total carbon number and *y* shows the location of double bonds [3]. Most of the microalgal phytosterols are in the free form with a relatively small number of conjugated forms. Conjugates are present as phytosterols with covalently bounded molecules particularly fatty acids and sugars at the OH group at C_3_ [4]. 

### 1.2. Origin and Applications of Phytosterols

There are more than 100 different types of phytosterols, which are under the triterpene family of nature products [5]. Cholesterol is the predominant sterol in animals whereas it is barely found in plants. Instead, plants contain several types of phytosterols, which are structurally similar and functionally analogous to cholesterols [6]. Phytosterols are present in all eukaryotic organisms, through either *de novo* synthesis or taken up from the environment [7]. They are important structural components of the cellular membrane and have important functions in regulating membrane fluidity and permeability. They also exist as hormones or hormonal precursors and are involved in signal transductions in the organisms [6]. 

Unlike cholesterol, humans cannot endogenously synthesise phytosterols and have to gain them from diet [8]. In the western diet, the average daily intake of phytosterols (mainly from vegetable oils, cereals, and fruits) is around 250 mg [9]. This amount of intake is estimated to be doubled for vegetarians [10]. Since the mid-1990s, phytosterol products have been commercialised as nutraceuticals or pharmaceuticals with the ability of lowering the blood cholesterol level, such as Cytellin marketed by Eli Lilly [11]. The main ingredient of Cytellin is sitosterol, which was used as either a supplement or as a drug for lowering cholesterol [12]. However, the market of phytosterols has not been revived until 1990s when Miettinen, Vanhanen [13] solved the issue of poor solubility and bioavailability of free phytosterols and achieved the consistency of the cholesterol-lowering effects with minimum amount of intake (2–3 g/day). Apart from its therapeutic values to treat hypercholesterolemia, phytosterols are also applied in other pharmaceutical areas as precursors of some bioactive molecules. An example is ergosterol as a precursor of vitamin D_2_ and an ingredient for producing cortisone and hormone flavone [14]. According to the Phytosterols Market Analysis by GVR [15], the global phytosterol demand was 49,299.6 tons (estimated revenue of USD 292.8 million) in 2013 and is expected to reach 80,535.9 tons (estimated revenue of 989.8 million) in 2020. Because of a forecasted increase in phytosterol market demand, alternative sources with high phytosterol content will generate great research and industry interest.

### 1.3. Health Promoting Effects of Phytosterol and Its Regulations

Phytosterols have received great attention because of its capacity of reducing the concentration of blood cholesterol and preventing the onset of cardiovascular disorders. In 2000, FDA issued an interim final rule authorising health claims for reduced risk of coronary heart disease (CHD) for phytosterol esters containing foods (65 FR54686) [16]. In 2010, FDA authorised the fortification of foods using nonesterified or free phytosterols. There are three phytosterols subject to the FDA health claim: β-sitosterol, campesterol and stigmasterol [17]. The Board of Food Standards Australia New Zealand (Proposal P1025) also gave notice of safe use of phytosterols and their esters in foods including breakfast cereals, cereal bars, milk and yoghurt. The European Atherosclerosis Society Consensus Panel approved the utilisation of phytosterol-enriched foods among patients with high cholesterol levels [18]. This favourable regulatory scenario is again going to further propel the global market growth of phytosterol and its products.

## 2. Microalgae as a Potential Source of Phytosterols

### 2.1. Types of Phytosterols from Microalgae for Human Consumption

To date, higher plants have been the main industrial sources of phytosterols [11], which are naturally present in vegetable oils, legumes, nuts, seeds, whole grains and dried fruits [4,19]. They are also found in algae: Chlorophyceae, Rhodophyceae and Phaeophyceae [6]. A standard reference regarding phytosterol content in different food sources can be found in United States Department of Agriculture’s National Nutrient Database. Phytosterol contents varied from 8.09 to 15.57 g per kg (equivalent to 0.809%–1.557% of oil weight) in corn oil, 19.7 g per kg (1.97%) in wheat germ oil and 32.25g per kg (3.225%) in rice bran oil [7]. In contrast, phytosterol content ranged from 7 to 34 g per kg (0.7%–3.4%) in four different microalgae oil extracts (*Isochrysis galbana*, *Nannochloropis gaditana*, *Nannochloropsis* sp. and *Phaeodactylum tricornutum*) depending on the solvent system being chosen [20]. More recently, *Pavlova lutheri*, *Tetrasellimis* sp. M8 and *Nannochloropsis* sp. BR2 were identified as the top three highest phytosterol producers (0.4%–2.6% dry weight) after screening hundreds of Australian isolates [21]. Research has reported that 5.1% dry weight of phytosterol could be achieved in *P. lutheri* by adjusting the nutrient, salinity and cultivation duration. Given that these phytosterol contents on the basis of microalgae dry biomass weight are equal or higher than all the plant oils extracted, it presents clear advantages to use microalgae directly as phytosterol supplements in various applications. The other two reasons that microalgae might be more advantageous to other common sources are their fast-growing characteristics and rich nutrient content [22]. The annual oil yield of some oil rich microalgal species varies from 19,000 to 57,000 L oil per acre, which is 60–200 times higher than the best-performing vegetable oils [23]. Some of them are also rich in other nutrients including proteins, carbohydrates, vitamins (vitamin A, B1, B2, B6, B12 and K, folate, niacin), minerals (calcium, phosphorous, iron, iodine, magnesium, zinc, selenium, copper, potassium, manganese and sodium) and various antioxidants (carotenoids, xanthophylls and chlorophyll) [24,25]. Many researches have reported the side effects of short and long term consumption of phytosterols as interfering with the absorption of β-carotenoid and vitamin E [4]. Therefore, it is suggested that dietary intake of these nutrients has to be increased at the same time to offset this absorption interference caused by phytosterol [26]. Microalgae may offer a complete and easy option due to its high vitamins, antioxidants and phytosterol contents and solve the issue of multi-supplementations for dietary needs.

In contrast with higher plants, there is a larger diversity of sterol distributions in microalgae [27] as listed in Table 1. Among microalgae, Glaucocystophyte are characterised by the presence of sitosterol and campesterol [28], Cyanobacteria by 24-ethylcholesterol [3], Cryptophytes by epibrassicasterol [29], Haptophytes by unusual dihydroxysterol from the genus *Pavlova* [30], Pelagophyceae by unusual 24-propylidenecholesterol mainly as the 24*E*-isomer [31], marine diatoms by 4-desmethyl-23,24-dimethyl steroid [32], Prasinophyceae by 24-methylenecholesterol and campesterol [33], Chlorarachniophyceae by crinosterol and stigmasterol [34], and most dinoflagellates by 4α-methyl sterols (especially dinosterol) [35] with the exception of Kareniaceae and *Polarella glacialis* [36]. 

Microalgae-derived phytosterols can be divided into four groups, 4-desmethyl-Δ^5^-sterols, 4-desmetyl-Δ^7^-sterols, 4-methyl sterols and dihydroxylated sterols [3]. The predominant phytosterol obtained from microalgae has Δ^5^ double bond and has no methyl groups at C_4_. Volkman (2003) summarised the occurrence of major sterols in different families of microalgae. Most sterols have 27 to 29 carbon atoms. Some species are exceptions. For example, the dinoflagellate *Prorocentrum* contains trace amount of 23 carbon sterol, the diatom *Chaetoceros* contains 26 carbon sterol, and Chrysophyte *Sarcinochrysis* and *Nematochrysopsis* contain 30 carbon sterol [37,38]. The composition of phytosterols varies depending on the strain and can be affected by factors such as light intensity, temperature and growth stage [39,40]. Some species such as dinoflagellates may contain a mixture of ten or even more sterol types [3]. With high phytosterol content in biomass and structure diversity between species, microalgae are promising sources of novel phytosterols with potential novel bioactivities. 

**Table 1:** Example of phytosterols identified from different microalgae species. Common names are used where applicable.

Species	Identified Phytosterols	References
*Attheya ussurensis* sp. nov.	24-Ethylcholest-5-en-3β-ol	[27]
*Bigelowiella*	Crinosterol, Stigmasterol	[34]
*Chattonella antique*	Isofucosterol	[41]
*Chattonella marina*	Isofucosterol	[41]
*Chattonella subsalsa*	Isofucosterol	[41]
*Chlorella vulgaris*	Ergosterol, 7-Dehydroporiferasterol, Ergosterol peroxide, 7-Oxocholesterol	[42]
*Chrysoderma* sp.	Stigmasterol, Sitosterol, Fucosterol	[38]
*Chrysomeris*	Stigmasterol, Sitosterol, Fucosterol	[38]
*Chrysowaernella*	Stigmasterol, Sitosterol, Fucosterol	[38]
*Crypthecodinium cohnii*	4α-Methyl sterols, Dinosterols, Dehydrodinosterol 4α,24-Dimethyl-cholestan-3β-ol 4α,24-Dimethylcholest-5-en-3β-ol Cholesta-5,7-dien-3β-ol	[43]
*Cyanophora paradoxa*	Sitosterol, Campesterol and 24-Ethylcholesta-5,22*E*-dien-3β-ol	[28]
*Diacronema vlkianum*	24-Ethylcholesta-5,22*E*-dien-3β-ol 4α-Methyl-24-ethyl-5α-cholest-22*E*-en-3β-ol	[30]
*Dunaliella salina*	Ergosterol, 7-Dehydroporiferasterol, 7-Dehydroporiferasterol peroxide, Ergosterol peroxide	[44,45]
*Dunaliella tertiolecta*	Ergosterol, 7-Dehydroporiferasterol	[44,46]
*Fragilaria pinnata*	23,24-Dimethylcholesta-5,22*E*-dien-3β-ol	[47]
*Giraudyopsis*	Stigmasterol, Sitosterol, Fucosterol	[38]
*Glaucocystis nostochinearum*	Sitosterol, Campesterol, 24-Ethylcholesta-5,22*E*-dien-3β-ol	[28]
*Gymnochlora*	Crinosterol, Stigmasterol	[34]
*Isochrysis galbana*	24-Oxocholesterol acetate, Ergost-5-en-3β-ol, Cholest-5-en-24-1,3-(acetyloxy)-,3β-ol	[48]
*Karenia brevis*	27-Nor-(24*R*)-4α-methyl-5α-ergosta-8(14),22-dien-3β-ol Brevesterol (24*S*)-4α-Methyl-5α-ergosta-8(14),22-dien-3β, its 27-Nor derivative	[49,50]
*Karenia mikimotoi*	27-Nor-(24*R*)-4α-methyl-5α-ergosta-8(14),22-dien-3β-ol Brevesterol, Gymnodinosterol (24*R*)-4α-Methyl-5α-ergosta-8(14),22-dien-3β-ol	[49]
*Karenia papilionacea*	23-Methyl-27-norergosta-8(14),22-dien-3β-ol	[49]
*Karenia umbella*	(24*R*)-4α-Methyl-5α-ergosta-8(14),22-dien-3β-ol Gymnodinosterol	[49]
*Karlodinium veneficum*	(24*R*)-4α-Methyl-5α-ergosta-8(14),22-dien-3β-ol Gymnodinosterol	[49]
*Lotharella*	Crinosterol and Stigmasterol	[34]
*Micromonas aff.pusilla*	24-Methycholesta-5,24(28)-dien-3β-ol 24-Methylcholesta-5-en-3β-ol 28-Isofucosterol and saringosterol	[51]
*Micromonas pusilla*	24-Methycholesta-5,24(28)-dien-3β-ol 24-Methylcholesta-5-en-3β-ol 28-Isofucosterol	[51]
*Navicula incerta*	Stigmasterol, 5β-Hydroxysitostanol	[52,53]
*Nematochrysopsis* sp.	(24*E*)-24-n-propylidenecholesterol	[38]
*Nitzschia closterium*	Cholesta-5,24-dien-3β-ol 24-Methylcholesta-5,22*E*-dien-3β-ol	[47]
*Nostoc commune var. sphaeroides Kützing*	Campesterol, Sitosterol, Clionasterol	[54,55]
*Olisthodiscus luteus*	Brassicasterol, Stigmasterol, Fucosterol	[41]
*Pavlova*	24-Ethylcholesta-5,22*E*-dien-3β-ol 4α-Methyl-24-ethyl-5α-cholest-22*E*-en-3β-ol	[30]
*Phaeodactylum tricornutum*	(24*S*)-24-Methylcholesta-5,22*E*-dien-3β-ol	[39]
*Polarella glacialis*	27-Nor-24-Methylcholest-5,22*E*-dien-3β-ol	[35]
*Porphyridium cruentum*	Stigmasterol, β-Sitosterol	[56]
*Pycnococcus provasolii*	24-Methycholesta-5,24(28)-dien-3β-ol 24-Methylcholesta-5-en-3β-ol 28-Isofucosterol	[51]
*Pyramimonas cf. cordata*	Stigmasterol	[27]
*Pyramimonas cordata*	24-Methycholesta-5,24(28)-dien-3β-ol 24-Methylcholesta-5-en-3β-ol 28-Isofucosterol	[51]
*Rhizosolenia setigera*	Cholesta-5,24-dien-3β-ol	[47]
*Sarcinochrysis* sp.	(24*E*)-24-n-propylidenecholesterol	[38]
*Schizochytrium aggregatum*	Campesterol, 24-Methylene cholesterol, Ergosterol, 24-Methyl-colest-7-en-3β-ol, Stigmasterol and others	[57]
*Schizochytrium* sp.	Lathosterol, Ergosterol, Stigmasterol, 24-Ethylcholesta-5,7,22-trienol, Stigmasta-7,24-(24^1^)-dien-3β-ol,	[58]
*Stephanodiscus meyerii*	24-Methycholesta-5,24(28)-dien-3β-ol	[27]
*Takayama helix*	27-Nor-(24*R*)-4α-methyl-5α-ergosta-8(14),22-dien-3β-ol Brevesterol	[49]
*Takayama tasmanica*	27-Nor-(24*R*)-4α-methyl-5α-ergosta-8(14),22-dien-3β-ol Brevesterol	[49]
*Tetraselmis chui*	24-Methycholesta-5,24(28)-dien-3β-ol 24-Methylcholesta-5-en-3β-ol 28-Isofucosterol	[51]
*Tetraselmis suecica*	24-Methylcholest-5-en-3β-ol 24-Methylcholest-5,24(28)-dien-3β-ol	[39]
*Thalassi-onema nitzschioides*	23-Methylcholesta-5,22*E*-dien-3β-ol 23-Methyl-5α-cholest-22*E*-en-3β-ol	[47]

### 2.2. Biosynthesis of Phytosterols in Microalgae 

The occurrence of sterols varies among plants, microorganisms, prokaryotes, yeasts and algae [59]. Similar to other organisms, microalgal phytosterols are also the end products of isoprenoid biosynthesis, from isopentenyl diphosphate (IPP) and dimethylallyl diphosphate (DMAPP) to squalene. Some microalgae retain two distinct and compartmentalised pathways for isoprenoid synthesis, mevalonic acid (MVA) pathway in the cytosol and the methyl-d-erythritol 4-phosphate (MEP) pathway in the plastid [7]. These microalgae arise from secondary endosymbiosis including Euglenophyta, Chlorarachniophyta, Heterokontophyta, Bacillariophyta and Haptophyta [59]. Some using both pathways may also arise from primary endosymbiosis such as Glaucophyta [60,61]. Exceptions are Prasinophyta and Chlorophyta, arising from primary endosymbiosis. They have completely lost the MVA pathway and exclusively produce sterols from MEP pathway. The exceptions within these two families are microalgae *Galdieria sulphuraria* [62] and *Cyanidium caldarium* [63], both of which use MVA pathway. A comprehensive review on the distribution of pathways of different microalgae families can be found by Lohr, Schwender [60]. The side chain of microalgal phytosterol contains an alkyl substitution at C_24_, which is added by sterol methyltransferase (SMT) in a step other than MEP or MVA pathways. This pathway is not necessary for the biosynthesis of some 27 carbon sterols [6]. 

### 2.3. Bio-Functionalities of Microalgal Phytosterols and Their Mechanisms of Action 

Phytosterols have been reported to have many beneficial health effects in humans, including immunomodulatory [46], anti-inflammatory [42,46], antihypercholesterolemic [54,58], antioxidant [64], anticancer [65,66] and antidiabetic [67]. Table 2 summarises the microalgal phytosterols undergoing functional tests. As shown, even though microalgae-derived phytosterols are diverse, limited studies have addressed their health-promoting activities.

#### 2.3.1. Cholesterol-Lowering Activity

Many studies have reported the cholesterol-lowering activity of consuming phytosterols and their esters, with 10%–15% reduction of low density lipoprotein serum cholesterol (LDL-C: major risk factors for CHD) shown among individuals with hypercholesterolemia [4]. The reduction was even more outstanding among patients who have been put on anti-hypercholesterolemic drugs such as statins [68] and fibrates [69]. The cholesterol-lowering activity was also observed for microalgae-derived phytosterols, which functioned by decreasing the dietary cholesterol absorption and endogenously-produced cholesterols from the gastrointestinal tract [58]. 

*Schizochytrium* sterol extract down-regulated the expression of intestinal gene *ACAT2* [58], which is responsible for cholesterol absorption in the intestine [70]. The cholesterol-lowering ability of this extract is also related to the down-regulation of hepatic 3-hydroxy-3-methylglutaryl-CoA (HMG-CoA) reductase, which is an enzyme involved in the synthesis of cholesterol. Meanwhile, *Schizochytrium* sterols stimulated the LDL-C receptor that facilitates the removal of plasma cholesterol from the circulation. Research showed that hamsters being fed on 0.06 and 0.3 g of *Schizochytrium* sterol extract per kg diet demonstrated a reduction of cholesterol level by 19.5% and 34%, respectively. The bioactivity of *Schizochytrium* sterol extract was as effective as the positive control group supplied with β-sitosterol, which is a phytosterol already added to food products, such as margarine and vegetable oils as healthy supplements. The mechanisms of action among different phytosterols are not the same. The lipid extract of blue-green alga, *Nostoc commune var. sphaeroides Kützing* (*N. commune*) has an inhibitory effect in cholesterol synthesis on human hepatoma cell lines by reducing the mRNA expression of 3-hydroxy-3-methylglutaryl-CoA reductase (HMGR) and LDL receptor [54]. The lipid of interest was not identified in this research; however, a previous study has reported that this species was characterized by the presence of campesterol, β-sitosterol and clionasterol [71]. Cholesterol-lowering activity of β-sitosterol is achieved by competing with cholesterol for transporter NPC1L1 as well as down-regulating its mRNA expression in the gastrointestinal tract [58]. β-Sitosterol are also found in some microalgae species (Table 3), however with their activity yet to be determined. As shown, some microalgae species have demonstrated a great potential in lowering plasma cholesterol and should be further explored. 

#### 2.3.2. Anti-Inflammatory Activity

Ergosterol isolated from edible mushrooms has the ability to suppress LPS-induced inflammatory responses of RAW264.7 macrophages *in vitro* through the inhibition of highly proinflammatory cytokine (TNF-α) production and COX-2 expression [72]. Ergosterol was found in *Chlorella vulgaris* [42] and *Dunalliella tertiolecta*, which demonstrated a similar mechanism of action via the reduction of LPS-induced response [46]. Apart from ergosterol, ergosterol peroxide, 7-dehydroporiferasterol peroxide and 7-oxocholesterol from *Chlorella vulgaris* also showed effective anti-inflammatory activities on 12-*O*-tetradecanoylphorbol-13-acetate (TPA: a potent tumour promoter)-induced mice with 0.2–0.7 mg/ear as 50% inhibitory dose [42]. The microalga *Dunaliella tertiolecta* has also been recently identified with ergosterol, 7-dehydroporiferasterol and ergosterol peroxide, and considered as future commercial source of phytosterol [44,45]. Some research reported a synergistic mechanism of some microalgal phytosterols regarding the enhancement of the bioactivity of another phytosterol. For example, the mixture of 7-dehydroporiferasterol with ergosterol (both from *Dunalliella tertiolecta*) further suppressed the proliferation of concanavalin A (ConA)-stimulated ovine peripheral blood mononuclear cells (PBMCs) compared with ergosterol alone at the same concentration [46]. Microalgal phytosterols and their secondary metabolites are promising potential anti-inflammatory agents and the synergistic effect should always take into consideration when optimising the functionality. 

#### 2.3.3. Anticancer Activity

Several studies have reported that phytosterols may have bioactivities against tumours [73]. For example, ergosterol showed cytostatic effect on human colorectal adenocarcinoma cells [72]. Ergosterol peroxide showed inhibitory effect on the growth of MCF-7 human mammary adenocarinoma and Walker 256 carcinosarcoma cells *in vitro* [74]. 2 μmol ergosterol peroxide from *Chlorella vulgaris* remarkably inhibited (77% reduction) the tumour progression by TPA and 7,12-dimethylbenz[*a*]anthracene (DMBA: immunosuppressor and tumour initiator)-initiated mice [42]. It was suggested that these bioactive sterols were functioned by inhibiting the accumulation of ornithine decarboxylase (ODC), which is a polyamine biosynthetic enzyme induced by TPA treatment. Stigmasterol isolated from *Navicula incerta* showed a significant toxicity on hepatocarcinoma (HepG2) cells in a dose-dependent manner and are effective to induce apoptosis via the up-regulation of pro-apoptotic gene *Bax* and *p53* and down-regulation of the anti-apoptotic gene *Bcl-2* [52,53]. Fucosterols and oxygenated fucosterol isolated from brown alga *Sargassum carpophyllum* exhibited cytotoxicity against different cancer cell lines [65,75]. The same phytosterols have also been identified in microalgae *Chrysoderma* sp. and *Olisthodiscus luteus* (Table 3); however, with functionality undetermined. Collectively, microalgae are promising resource for chemopreventive agents in cancer therapy but further studies are required to identify the equality between the phytosterols of interest and those derived from microalgae species.

#### 2.3.4. Antioxidant 

Evidence of antioxidant activity from microalgae-derived phytosterols was in scarcity. However, phytosterols derived from other resources have been reported showing positive effects. For instance, stigmasterol derived from the bark of *Butea monosperma* showed highest pro-oxidative dose of 5.2 mg per kg of food intake per day by reducing the tissue lipid peroxidation (major cause of cellular damage) and increasing the activities of catalase, superoxide dismutase (SOD) and glutathione, which are endogenous antioxidants [76]. In addition, carbon tetrachloride (CCl_4_)-intoxicated rats, undergoing treatment with 30 mg (fucosterol derived from marine algae *Pelvetia siliquosa*) per kg of food intake per day for seven consecutive days, showed significant decrease of serum transaminase activities and increase of free radical scavenging enzymes such as SOD, catalase and glutathione peroxidise by 33.89%, 21.56% and 39.24%, respectively [64]. Even though fucosterols have been found in several microalgae species such as *Chrysoderma* sp*.*, *Chrysomeris*, *Chrysowaernella* and *Giraudyopsis* [38], no research could be found specifically analysing the activity of microalgae-derived fucosterols. Lipid extracts of some microalgae species have been identified with antioxidant activity, such as *Schizochytrium aggregatum* [57], however, the compounds of interest remain to be determined. This identification is urgently required, because of the health concern and risks caused by synthetic antioxidants in the market such as butylated hydroxytoluen (BHT) and propyl gallate (PG) used in food and pharmaceutical industries [77]. Microalgal phytosterols as natural products are more preferable alternatives for antioxidants for human consumption.

#### 2.3.5. Other Activities

Other activities relating to microalgae-derived phytosterols include antibacterial and antidiabetic activities. Tuberculosis is the second most common cause of human death, and it is contagious and airborne [78]. Prakash, Sasikala [48] found that the extract of microalgae *Isochrysis galbana* (with 24-oxocholesterol acetate, ergost-5-en-3β-ol and cholest-5-en-24-1,3-(acetyloxy)-,3β-ol) at 50 μg per mL inhibited multidrug resistant *Mycobacterium tuberculosis* compared to the tuberculosis drug amikacin at 700 μg per mL, pyrazinamide at 200 μg per mL and rifambicin at 40 μg per mL. Furthermore, 0.5 μg per mL saringosterol isolated from brown algae *Sargassum ringgoldianum,* could also inhibit the growth of *M. tuberculosis* H_37_Rv, which has been found to be as efficient as tuberculosis drug rifampicin in the same assay [79]. Saringosterol has also been found in microalgae *Micromonas aff. pusilla* [51] but with undefined bioactivity. Diabetes is a chronic disease characterised by high blood glucose level and acute complications such as hypoglycaemia. Fucosterols isolated from the seaweed *Pelvetia siliquosa* have been identified with anti-diabetic activity in streptozotocin-induced diabetic rats [67]. Several microalgae species have also been reported producing fucosterols such as *Chrysoderma* sp. and *Olisthodiscus luteus* [38,41]; however, the functionality of these fucosterol are remained to be tested. Due to the high annual yield of microalgae lipids, microalgae-derived phytosterols as natural products [44] have a much greater potential to the drug industry; which may not only solve the issue of the upcoming surge of the global demand but also offer more preferable alternatives due to side effects associated with synthetic drugs.

**Table 2:** Bioactivities of phytosterols derived from microalgae. Abbreviation: DPPH: 2, 2-diphenyl-1-picrylhydrazyl; HMGR: 3-hydroxy-3-methylglutaryl-CoA reductase; SREBP-1: sterol regulatory element binding protein -1.

Microalgae Species	Major Phytosterols	Biological Activity	Function	References
*Chlorella vulgaris*	Ergosterol, 7-Dehydroporiferasterol, Ergosterol peroxide, 7-Dehydroporiferasterol peroxide, 7-oxocholesterol	Anti-inflammatory	50% inhibitory dose was 0.2–0.7 mg/ear	[42]
*Chlorella vulgaris*	Ergosterol peroxide	Anti-cancer	2 μmol led to 77% reduction in tumour progression	[42]
*Dunaliella tertiolecta*	Ergosterol, 7-Dehydroporiferasterol	Immunomodulatory Anti-inflammatory	0.4 mg/mL mixture for the highest production of IL-10, 0.8mg/mL for ergosterol alone	[46]
*Dunaliella tertiolecta*	Ergosterol, 7-Dehydroporiferasterol	Neuromodulatory	Neuromodulatory action was found in selective brain areas of rats	[80]
*Isochrysis galbana*	24-Oxocholesterol acetate, Ergost-5-en-3β-ol, Cholest-5-en-24-1,3-(acetyloxy)-, 3β-ol and others	Antituberculosis	Minimum inhibitory concentration of 50–60 μg/mL against *M. tuberculosis*	[48]
*Navicula incerta*	Stigmasterol, 5β-Hydroxysitostanol	Anti-cancer	40%, 43% and 54% toxicity at 5, 10 and 20 μM, respectively	[52,53]
*Nostoc commune var. sphaeroides Kützing*	Lipid extract	Cholesterol-lowering activity	Reduced HMGR activity by 90% and reduced SREBP-1 mature protein by 30%	[54]
*Schizochytrium aggregatum*	Campesterol, 24-Methylene cholesterol, 24-Methyl-colest-7-en-3β-ol, Ergosterol, Stigmasterol and other lipids	Antioxidant	IC_50_ in DPPH radical scavenging study was 5.76 mg/mL. Digested microalgae oil had an α-tocopherol equivalent antioxidant capacity of 42.071 μg/mg At 10 mg/mL, reducing power was 0.874	[57]
*Schizochytrium* sp.	Lathosterol, Ergosterol, Stigmasterol, 24-Ethylcholesta-5,7,22-trienol, Stigmasta-7,24-(24^1^)-dien-3β-ol and others	Cholesterol-lowering activity	0.06–0.3 g/kg diet decreased blood cholesterol by 19.5%–34%	[58]

**Table 3:** Functional phytosterols observed in microalgae and the original resources for the function identification. Information on nomenclatures gathered from [4] with adaptations.

	Chemical Structure	Nomenclatures	Species of Origin	Bioactivity	Same Sterol(s) Observed in Microalgae
Campesterol	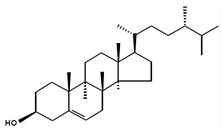	Campesterin Cholest5-en-3-ol (24*R*)-24-Methylcholest-5-en-3β-ol Campest-5-en-3β-ol Δ^5^-24α-Methyl-cholesten-3β-ol (24*R*)-Ergost-5-en-3β-ol	Flower *Chrysanthemum coronarium* L. [66] Red algae *Porphyra dentata* [81] *Shorea singkawang* [82]	Cholesterol-lowering Anticancer Antiangiogenic	*Tetraselmis* [33] *Porphyridium cruentum* [83] *Schizochytrium aggregatum* [57]
7-Dehydroporiferasterol	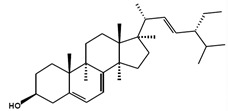	(22*E*,24*R*)-Ethylcholesta-5,7,22-trien-3β-ol 24*R*-Stigmasta-5,7,22-trien-3β-ol Porifersta-5,7,22*E*-trienol	Rarely found in other organisms	-	*Chlorella vulgaris* [84] *Chlamydomonas reinhardtii* [85] *Dictyonella incisa* [86]
Ergosterol	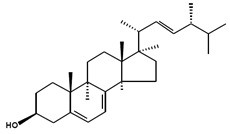	(22*E*)-Ergosta-5,7,22-trien-3β-ol (22*E*,24*R*)-Methylcholesta-5,7,22-trien-3β-ol	Mushroom *Sarcodon aspratus* [72] Mushroom *Inonotus obliquus* [87] *Ganoderma lucidum* [88] *Agaricus bisporus* [89]	Anticancer Anti-inflammatory Cholesterol-lowering	*Chlorella pyranoidosa* [90] *Dunaliella tertiolecta* [80] *Schizochytrium aggregatum* [57]
Fucosterol	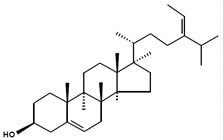	(24(28)*E*)-Stigmasta-5,24(28)-dien-3β-ol (24(24′)*E*)-Stigmasta-5,24(24’)-dien-3β-ol (24*E*)-Ethylidenecholesta-5,24(28)-dien-3β-ol	Macroalgae *Pelvetia siliquosa* [64,67] Brown alga *Turbinaria conoides* [65] Macroalgae *Himanthalia elongate*, *Undaria pinnatifida*, *Phorphyra* sp., *Chondus crispus*, *Cystoseira* sp. and *Ulva* sp. [91]	Antioxidant Antidiabetic Anticancer Cholesterol-lowering	*Chrysoderma* sp. *Chrysomeris* *Chrysowaernella* *Giraudyopsis* [38] *Olisthodiscus luteus* [41]
Saringosterol	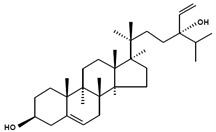	24(*S*)-Saringosterol Sargasso sterol	Brown algae *Sargassum ringgoldianum* [79] *Sargassum thunbergii* [92] *Lessonia nigrescens* [93] Seaweed *Sargassum fusiforme* [94]	Antitubercular Antiatherosclerotic Lipase-inhibitory	*Micromonas aff.pusilla* [51]
β-Sitosterol	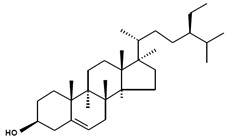	Sitosterol Stigmast-5-en-3β-ol 24α-Ethylcholest-5-en-3β-ol	Peanuts [95] Coral *subergorgia reticulate* [96] Plant *Verbena officinalis* [97] Leaves of *Mentha cordifolia* Opiz [98]	Anticancer Anti-inflammatory Analgesic activity Anthelminthic Antimutagenic	*Bigelowiella natans* *Gymnochlora stellata* *Lotharella amoeboformis* [34] *Porphyridium cruentum* [56]
Stigmasterol	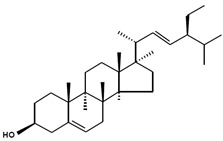	(Δ^5,22*E*^) (24α=24*S*) Poriferasterol (22*E*)-Stigmasta-5,22-dien-3β-ol 24α-Ethylcholesta-5,22*E*-dien-3β-ol	*Butea monosperma* [76] *Parkia speciosa* seeds [99]	Thyroid-inhibitory Antioxidant Hypoglycaemic	*Porphyridium cruentum* [56]
Δ^5^-Avenasterol	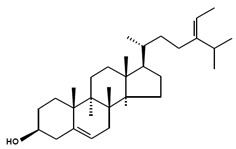	(5-Avenasterol) (Δ^5,24Z^) Isofucosterol 28-Isofucosterol 29-Iso-fucosterol 24*Z*-Ethylidenecholesta-5,24(28)-dien-3β-ol [24(28)*Z*]-Stigmasta-5,24(28)-dien-3β-ol [24(24′)*Z*]-Stigmasta-5,24(28’)-dien-3β-ol	Brown algae *Fucus vesiculosus* Green algae *Ulva lactuca* [100] Wheat germ oil [101] Tomato seed oil [102] Sargassum thunbergii [92] Rape bee pollen [103] Marine sponge *Petrosia weinbergi* [104]	Antioxidant Lipase-inhibitory Precursor of antiviral orthoesterol	Myxophyceae Chlorophyceae [55] *Chattonella marina* [41]
Brassicasterol	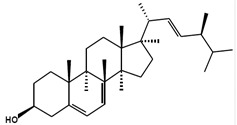	24-Methyl cholest-5,22-dien-3β-ol (3β,22*E*)-Ergosta-5,22-dien-3-ol Ergosta-5,22-dien-3β-ol	Rapeseed oil [105]	Cholesterol-lowering	*Isochrysis galbana* and *Chaetoceros calcitrans* [106] *Rhodomonas salina* [107]

### 2.4. Advanced Green Extraction and Purification Technology of Phytosterols 

The industry-scale technology for phytosterol recovery utilises sterol containing materials such as tall oils or vegetable oils and is processed by hydrolysation of the steryl esters into free sterols [11]. This process is rather complex, energy intensive and involves organic solvents and toxic chemicals, such as chloroform, methanol, hexane and sodium hydroxide [108,109,110,111]. The same extraction process was also found when extracting phytosterols from *D. tertiolecta*, *D. salina* [44] and *Pyramimonas cf. cordata* [27]. Even though substantial yield of phytosterols might be achieved, the application of toxic chemicals may hamper its application in food and pharmaceutical industry. 

Methods for green extraction technology of phytosterols, include supercritical carbon dioxide extraction (SC-CO_2_) [112,113,114,115,116,117]. Carbon dioxide has been used as the first choice solvent in more than 90% of the supercritical fluid extraction of bioactive compounds from natural resources [118], due to the benefits of safe, inexpensive, recyclable and being non-hazardous to health and environment [119]. Optimization of extraction parameters such as pressure, temperature, flow rate of CO_2_ was required when perform SC-CO_2_. This processing technology applies to various sources and is considered to be an effective and environmentally friendly technique for the separation of solvent-free phytosterols [120]. SC-CO_2_ has also been applied for general microalgal lipid production [121,122]; however, it has been rarely integrated with high performance liquid chromatography (HPLC) or gas chromatography-mass spectrometry (GC-MS) to specifically analyze the phytosterol component. SC-CO_2_ is a clean and food safety-guaranteed extraction method. Future research should investigate more into applying this technology to phytosterol production from microalgae biomass. 

## 3. Future Prospects of Microalgae-Derived Phytosterols

One of the limitations in the development of microalgae-derived phytosterols is their low sterol content [3,44]. Compared with other commercial plant sources, some microalgae species show an equivalent phytosterol content and this figure may surpass that found in some conventional sources when choosing the best performing microalgae strain with optimised cultivation conditions. In recent years, phytosterols from microalgae are starting to attract more attention due to the diversity of phytosterols in these species. The utilisation of microalgae for phytosterols production offers an opportunity for finding novel phytosterols with potential benefits to human health or a mixture of molecules able to synergistically enhance the bioactivity of a single phytosterol [46]. 7-Dehydroporiferasterol acting as a good example of phytosterol derived from microalgae with outstanding anti-inflammatory activities but has rarely been observed in other organisms. Over the last decade, researchers have started to analyse the bioactive phytosterols isolated from macroalgae [65,75,123,124,125] but studies on microalgal sterols have lagged far away behind. Most microalgal sterol research was conducted on the analysis and characterisation of sterol components within different species (Table 1) but with very limited amount of studies focusing on the bioactivities and functionalities of those sterols (Table 2). For the studies with identified bioactivities, further endeavours should be aimed at identifying the sterol species responsible for the activity. 

The screening of microalgal sterols for bioactivity could be directed by the chemical structure of the sterol of interest. A close dependency between the skeletal structure of sterols and their bioactivity has been reported as evidenced by the remarkable anticancer activity within groups of Δ^5,7^-sterol, 5α,8α-epidioxy-Δ^6^-sterols and 7-oxo-Δ^5^-sterol [42]. It was suggested that the double bonds at C_5_ and C_22_ in phytosterols are responsible for the apoptosis induction effect [52]. In the same vein, Hernandez-Ledesma, Blanca [6] and Nes [126] reported that rings A and D are of particular importance to sterol’s function and the stereochemistry of the C_24_ alkyl group is the key to intermolecular interactions. This importance on sterol structure is also proved by the addition of an oxo group at C_7_ of cholesterol, a Δ^5^-sterol, which showed poor inflammatory activity on its own. However, after the addition of oxo group, the 7-oxo-Δ^5^-sterol considerably increased the anti-inflammatory effect on TPA-induced inflammatory mice [42]. The same theory also applied to Δ-5-avenasterol which showed antioxidant activity due to the presence of ethyliden group in 24, 28 position of the R chain [102]. Microalgal species rich in docosahexaenoic acid (DHA) and docosapentaenoic acid (DPA) may also offer a clue for the screening process. Previous studies on DHA and DPA derived from *Schizochytrium* sp. were identified with cholesterol-lowering activity through the down-regulation of HMG-CoA reductase [127]. The later study observed that the sterols derived from this species also contributed to the activity [58]. 

Given microalgal phytosterols are diverse, it is essential to differentiate the potential of the different types of sterols in microalgae, including their mechanism of actions, synergic effects with other compounds and the effects of long-term treatment. To emphasise that, the same sterols isolated from different microalgae species might be in a mixture of epimers [93]. The function of the epimers should always be questioned compared to the pure ones. This is because sargosterol (mixture of 24S and 24R epimers) isolated from *Lessonia nigrescens* were eight times more active against *M. tuberculosis* H_37_Rv than 24S isomer alone [93]. Even though microalgae-derived phytosterols have rarely been reported with toxicity, some microalgae like Dinophyceae class may produce toxins especially during harmful algal blooms [128]. Bioactive phytosterol extracts should also undergo toxicity assays (organisms-based or cell line-based) and chemical analysis (such as LC-MS) to verify their applicability in food and pharmaceutical industries [129]. Elimination of toxins, such as okadaic acid, dinophysistoxins and brevetoxins, is compulsory before further processing. In addition, phytosterol-fortified foods are rich in free phytosterols and their fatty acid esters, which are susceptible to oxidation, future research should also investigate the stability of microalgal phytosterol when applied to food fortification. The compounds resulting from phytosterol oxidation could exert toxic effects and initiate the major chronic diseases [130]. This could be done by the analysis of the production of phytosterol oxidation products (POPs). To improve the oxidative stability, phytosterols could be incorporated to a matrix with natural antioxidant compounds such as milk based fruit beverages [131].

## 4. Conclusions

Phytosterols have grown in popularity due to their health-promoting activities over the past few decades. New sources are urgently needed to meet the growing demand of phytosterols for functional food and pharmaceutical industries. Microalgae as one of the best alternatives could offer different types of phytosterols and other high-valued compounds at a much higher efficiency than terrestrial plants. However, the research on microalgal phytosterols is mainly focusing on the area of analysing and identifying sterol constituents with most of their bioactivities unknown. Thus, research in the future should focus more on the functional activity of microalgae-derived phytosterols and their applications in food and pharmaceutical industries. 
